# Medication Nonadherence and Risk of Violence to Others Among Patients With Schizophrenia in Western China

**DOI:** 10.1001/jamanetworkopen.2023.5891

**Published:** 2023-04-05

**Authors:** Yang Li, Hong Wen, Chaoxinyu Xiong, Chunying Lin, Xianmei Yang, Dan Wang, Ruoxing Fan, Jun Liu, Xing Zhao, Yuanyuan Liu, Xiang Liu

**Affiliations:** 1 Department of Epidemiology and Biostatistics, West China School of Public Health and West China Fourth Hospital, Sichuan University, Chengdu, China; 2 Sichuan Mental Health Center, Third Hospital of Mianyang, Mianyang, China; 3 Fuwai Hospital, National Center for Cardiovascular Diseases, Chinese Academy of Medical Sciences and Peking Union Medical College, Beijing, China; 4 Department of Health Behavior and Social Medicine, West China School of Public Health and West China Fourth Hospital, Sichuan University, Chengdu, China

## Abstract

**Importance:**

Reducing violence to others in community-based patients with schizophrenia has important implications for public health. Increasing medication adherence is often used to reduce the risk of violence, yet little is known about the association between medication nonadherence and violence to others in this population.

**Objective:**

To examine the association between medication nonadherence and violence to others among community-based patients with schizophrenia.

**Design, Setting, and Participants:**

This large, naturalistic, prospective cohort study was performed in western China from May 1, 2006, to December 31, 2018. The data set was from the integrated management information platform for severe mental disorders. As of December 31, 2018, 292 667 patients with schizophrenia were registered in the platform. During follow-up, patients could enter or leave the cohort at any time. Maximum follow-up was 12.8 years, with a mean (SD) of 4.2 (2.3) years. Data analysis was conducted from July 1, 2021, to September 30, 2022.

**Exposures:**

Medication nonadherence.

**Main Outcomes and Measures:**

Violence to others throughout the follow-up period was the outcome, including minor nuisances, violating the Law of the People’s Republic of China on Penalties for Administration of Public Security (APS law), and violating criminal law. Information about these behaviors was provided by the public security department. Directed acyclic graphs were used to identify and control confounders. Propensity score matching and generalized linear mixed-effects models were used for analysis.

**Results:**

The final study sample included 207 569 patients with schizophrenia. The mean (SD) age was 51.3 (14.5) years, and 107 271 (51.7%) were women; 27 698 (13.3%) perpetrated violence to others, including 22 312 of 142 394 with medication nonadherence (15.7%) and 5386 of 65 175 with adherence (8.3%). In 112 710 propensity score–matched cases, risks of minor nuisances (odds ratio [OR], 1.82 [95% CI, 1.75-1.90]; *P* < .001), violating APS law (OR, 1.91 [95% CI, 1.78-2.05]; *P* < .001), and violating criminal law (OR, 1.50 [95% CI, 1.33-1.71]; *P* < .001) were higher in patients with nonadherence. However, the risk did not increase with higher medication nonadherence. There were differences in risk of violating APS law between urban and rural areas.

**Conclusions and Relevance:**

Medication nonadherence was associated with a higher risk of violence to others among community-based patients with schizophrenia, but the risk did not increase as medication nonadherence increased.

## Key Points

**Question:**

Is medication nonadherence associated with risk of violence to others among patients with schizophrenia?

**Findings:**

In this cohort study of 207 569 community-based patients with schizophrenia, nonadherence to medication was associated with higher risk of violence in the form of minor nuisances, violating public security law, and violating criminal law. The risk of violence did not increase with higher medication nonadherence.

**Meaning:**

These findings suggest that medication nonadherence may be associated with the increased risk of violence to others among community-based patients with schizophrenia.

## Teaser

This cohort study explores the association between medication nonadherence and violence to others among community-based patients with schizophrenia in China.

## Introduction

Schizophrenia is a severe mental disorder affecting 20 million people worldwide.^1^ More than one-quarter of patients with schizophrenia are from China,^2^ and the number of cases is increasing every year.^3^ Some studies have suggested that patients with schizophrenia are more likely than the general population to commit violent behaviors.^4^ The occurrence of violent behaviors seriously affects the quality of life of patients and their families and has a negative impact on social security. How to effectively reduce violent behaviors among patients with schizophrenia is an important issue that deserves in-depth study.

The current treatment of schizophrenia in China is dominated by medication. The use of antipsychotics is considered the cornerstone of treatment for schizophrenia and can effectively reduce the incidence of violent behaviors among patients.^5^ However, medication nonadherence is a prevalent problem in patients with schizophrenia, with the nonadherence rate ranging from 40% to 60%.^6^ Research has been conducted on the effects of medication nonadherence on violent behaviors among patients with schizophrenia, but the following problems still exist: (1) most studies have focused on the correlation of factors influencing violent behaviors,^7^ and few have focused on the exploration of medication nonadherence; (2) the research methods were mostly traditional statistical methods^8,9,10^ without rigorous analysis techniques, especially for the identification and control of confounders; (3) the study types were mainly cross-sectional surveys and retrospective studies^8,9,11^ with a lack of prospective studies, especially those with large sample sizes; and (4) the study participants were mainly inpatients^8,9,11^ with a lack of studies that included community-based patients.

Considering that most patients with schizophrenia in China do not receive long-term systematic inpatient treatment and are mainly living in the community, this study explored the association between medication nonadherence and violence to others, including minor nuisances, violating the Law of the People’s Republic of China on Penalties for Administration of Public Security (hereinafter APS law), and violating criminal law^1^ based on a large, 12-year prospective cohort. We hypothesized that (1) patients with medication nonadherence would be more likely to commit violence to others than those with better medication adherence, using 80% adherence as the threshold, and (2) the risk of violence to others would increase concomitantly with medication nonadherence compared with patients with high medication adherence (≥80%). To our knowledge, this is the first study focusing on the association between medication nonadherence and violence to others in community-based patients with schizophrenia, and the findings may fill the gap in the related field and provide a reference for future community violence control and prevention.

## Methods

### Study Design and Study Population

This cohort study was performed in accordance with International Ethical Guidelines on Biomedical Research Involving Human Subjects and the Declaration of Helsinki. This study received institutional review board approval for research involving human participants from Sichuan University and Sichuan Mental Health Center. Patients and/or guardians were consulted and signed the informed consent forms to participate in community case management and treatment services for severe mental disorders. Data were recorded as a part of routine management, but all data that potentially allow for the identification of individuals were anonymized before the data analysis. This study followed the Strengthening the Reporting of Observational Studies in Epidemiology (STROBE) reporting guideline.

The data set was from the integrated management information platform for severe mental disorders (hereinafter referred to as the platform) in western China. The platform launched a pilot trial in 2006, which was scaled up in practice by 2010. The platform was managed by professionals at mental health centers and the Centers for Disease Control and Prevention. Primarily, the platform used 2 approaches to engage patients. Initially, they identified patients referred from related departments in general hospitals or psychiatric hospitals. Second, they screened patients referred from the China Disabled Persons’ Federation, township health centers, local communities, neighborhoods, or village committees for possible psychosis. These patients were then examined by psychiatrists, and those who met the diagnostic criteria of the *International Statistical Classification of Diseases and Related Health Problems, Tenth Revision* or *Diagnostic and Statistical Manual of Mental Disorders* (Fourth Edition)^12^ were evaluated for their disease condition by clinicians. This evaluation was focused on an assessment of risk for violence, social functionality, self-consciousness, mental symptoms, adverse drug reactions, and other serious physiological diseases.^3^ According to the relevant regulations, the frequency of evaluation in a follow-up depended on the patient’s disease condition. Patients with stable or basically stable disease were followed up at least once every 3 months. Patients with unstable disease status were followed up at least once every 2 weeks. From May 1, 2006, to December 31, 2018, 292 667 patients with schizophrenia were registered. During the follow-up period, patients could enter or leave the cohort at any time. Maximum follow-up reached 12.8 years, with a mean (SD) follow-up period of 4.2 (2.3) years. Among them, 239 402 (81.8%) patients underwent treatment in rural areas, whereas the remaining 53 265 (18.2%) patients resided in urban areas.

Since the number of follow-up records obtained varied depending on the time of entry into the cohort, in addition to the patients with missing records for violence to others or confounders, medication adherence, or medical advice, those who were not required to receive medication were excluded from this study, and thus a total of 207 569 patients were included in the analysis. The study selection flowchart is provided in eFigure 1 in Supplement 1.

### Outcomes

Violence to others was the outcome of interest. According to Work Specification for the Management and Treatment of Severe Mental Disorders,^13^ violence to others includes (1) minor nuisances (ie, instances in which the patient’s behavior does not constitute a violation of APS law; (2) violating APS law; and (3) violating the criminal law of the People’s Republic of China. These behaviors were recorded by clinicians during each follow-up period of patients according to the records provided by the public security department. For each patient, the number of occurrences of minor nuisances, violating APS law, and violating criminal law in the entire follow-up period was recorded. If the number of occurrences of violence to others was greater than 0, the patient was considered to have committed violence to others (please see the definitions and encoding in eTable 1 in Supplement 1).

### Exposure

Medication nonadherence was the exposure of interest in this study and refers to a patient’s adherence to psychotropic medication regimens throughout the follow-up period. According to the Work Specification for the Management and Treatment of Severe Mental Disorders,^13^ medication adherence is divided into 4 levels: (1) regular adherence, when patients take medications as prescribed throughout the follow-up period; (2) intermittent adherence, when patients fail to take medications as prescribed or as per the required frequency or quantity; (3) nonadherence, when patients do not take medications at all; (4) no medication, when no medication is required as prescribed by physicians (patients in such cases are excluded). Each time a patient is followed up, the patient’s medication adherence level is recorded for that follow-up period, so most patients had more than 1 adherence record. In the following analysis, we first calculated the proportion of regular medication (PRM; range, 0-1.00) during the follow-up period for each patient. Subsequently, we divided medication adherence into 2 groups by referring to the previous cutoff value of 0.80^14^ for nonadherence (PRM < 0.80) and adherence (PRM ≥ 0.80). This is the primary exposure variable in this study for the first hypothesis. In addition, we classified medication adherence into 5 classes according to the magnitude of PRM, where P1 (PRM range, 0 to <0.20), P2 (PRM range, 0.20 to <0.40), P3 (PRM range, 0.40 to <0.60), and P4 (PRM range, 0.60 to <0.80) are the medication nonadherence groups and P5 (PRM range, 0.80-1.00) is the medication adherence group. We further investigated whether increased medication nonadherence is associated with a higher risk of violence to others for the second hypothesis.

### Statistical Analysis

Data were analyzed from July 1, 2021, to September 30, 2022. First, to explore differences in the relevant influencing factors among patients with medication nonadherence or adherence, we conducted univariate analyses using *t* tests, χ^2^ tests, and standardized mean difference. Second, directed acyclic graphs (DAGs) were constructed representing associations among medication nonadherence, other confounders, and violence to others as stated in the existing literature.^4,7,15,16^ A minimally sufficient set of covariates was selected to identify confounders, according to the d-separation^17^ and the protocol of evidence synthesis for constructing DAGs.^18^ The independence of the DAGs was tested after removing unmeasured confounders and corrected to obtain the final DAGs.

Third, propensity score matching (PSM)^19^ and generalized linear mixed-effects models (GLMMs) were used to explore associations between medication nonadherence and violence to others. Since patients were not randomly allocated to the different medication adherence groups, associations between exposures and outcomes might be biased. The propensity scores of the patients reflect their probability of belonging to the medication nonadherence group, which was calculated based on logistic regression models that included the minimally sufficient set of covariates from DAGs and region (eTable 2 in Supplement 1). By matching patients from medication nonadherence and adherence groups based on propensity scores, data sets with similar distribution of confounders can be generated. In matching procedures, a 1:1 nearest-neighbor matching approach was implemented, and a caliper set at 0.2 SD of the logit of the propensity score was used (eMethods 2 in Supplement 1). Overall, 56 355 pairs of matched cases were created, which consisted of 112 710 cases and resulted in a good balance of confounders (eFigure 2 in Supplement 1). Density plots of the propensity scores before and after matching were compared (eFigure 3 in Supplement 1). As there was still a hierarchical structure of data after matching (eTable 4 in Supplement 1), GLMMs (binomial distribution) were fitted to examine whether patients with medication nonadherence differed from those with adherence with respect to the odds of violence to others (primary hypothesis). Models included random intercepts per region and per patient (nested within region) to account for the nonindependence of cases resulting from differences between regions. For the second hypothesis, both PSM and GLMMs were also used to examine the odds of violence to others across medication nonadherence levels (P1 to P4) compared with the medication adherence (reference) group (P5).

Fourth, subgroup analyses were performed. Stratification was first performed according to sex and urban or rural residence; then PSM was performed in each stratum to obtain matched samples by including confounders and region other than stratification factors; then GLMMs were used to analyze associations between exposures and outcomes. Last, sensitivity analyses were conducted using bounds methods developed by Rosenbaum and Rubin,^20^ which were to test the conditional independence hypothesis to determine the influence of unmeasured potential confounders in the DAG that were not collected in our database (eMethods 3 in Supplement 1).

The significance level was set at *P* < .05, and all *P* values were 2 sided. DAGs analysis was conducted using DAGitty software, version 3.0 for R (Tumor Immmunology Lab and Institute for Computing and Information Sciences, Radboud University Nijmegen).^21^ All other data analyses were performed using R, version 4.2.1 (package MatchIt,^22^ lme4, and rbounds; R Project for Statistical Computing).

## Results

Our sample included 207 569 community-based patients with schizophrenia. The mean (SD) age was 51.3 (14.5) years; 107 271 (51.7%) were women and 100 298 (48.3%) were men. A total of 27 698 individuals (13.3%) committed violence to others, including 25 428 (12.3%) minor nuisances, 7936 (3.8%) violations of APS law, and 1958 (0.9%) violations of criminal law. Medication nonadherence was found among 142 394 patients (68.6%) (eTable 3 in Supplement 1).

Table 1 shows the comparison of sociodemographic characteristics between patients who were medication nonadherent and adherent. Patients who were nonadherent tended to be older. Compared with the adherence group, a higher proportion of patients in the nonadherence group were members of ethnic minority populations, were married, lived in rural areas, had low levels of educational attainment, lived in poverty, and had longer follow-up time and duration of illness. There was no significant difference in sex between the 2 groups.

**Table 1.** Sociodemographic Characteristics of Study Patients

Characteristic	Total cohort (N = 207 569)	Patient group^a^		SMD	*P* value
		Medication nonadherence (n = 142 394)	Medication adherence (n = 65 175)		
Age, mean (SD), y	51.3 (14.5)	53.1 (14.2)	47.4 (14.1)	0.40	<.001^b^
Sex					
Men	100 298 (48.3)	68 916 (48.4)	31 382 (48.2)	0.01	0.30^c^
Women	107 271 (51.7)	73 478 (51.6)	33 793 (51.8)		
Ethnicity					
Han	205 045 (98.8)	140 540 (98.7)	64 505 (99.0)	0.03	<.001^c^
Ethnic minority	2524 (1.2)	1854 (1.3)	670 (1.0)		
Marital status					
Married	134 154 (64.6)	94 211 (66.2)	39 943 (61.3)	0.13	<.001^c^
Unmarried	73 415 (35.4)	48 183 (33.8)	25 232 (38.7)		
Urban and rural					
Rural	170 534 (82.2)	127 701 (89.7)	42 833 (65.7)	0.60	<.001^c^
Urban	37 035 (17.8)	14 693 (10.3)	22 342 (34.3)		
Educational attainment					
Primary school or lower	140 357 (67.6)	107 506 (75.5)	32 851 (50.4)	0.55	<.001^c^
Middle and high school	63 688 (30.7)	33 855 (23.8)	29 833 (45.8)		
College, university, or higher	3524 (1.7)	1033 (0.7)	2491 (3.8)		
Economic status					
Poverty^d^	135 868 (65.5)	100 086 (70.3)	35 782 (54.9)	0.32	<.001^c^
Nonpoverty	71 701 (34.5)	42 308 (29.7)	29 393 (45.1)		
Community case management status					
Yes	206 758 (99.6)	141 793 (99.6)	64 965 (99.7)	0.02	0.01^c^
No	811 (0.4)	601 (0.4)	210 (0.3)		
Follow-up time, y					
<2	47 329 (22.8)	24 942 (17.5)	22 387 (34.3)	0.41	<.001^c^
2-3	61 596 (29.7)	43 023 (30.2)	18 573 (28.5)		
≥4	98 644 (47.5)	74 429 (52.3)	24 215 (37.2)		
Duration of illness, y					
<10	72 962 (35.2)	45 678 (32.1)	27 284 (41.9)	0.24	<.001^c^
10-19	65 178 (31.4)	45 360 (31.9)	19 818 (30.4)		
20-29	39 775 (19.2)	28 271 (19.9)	11 504 (17.7)		
≥30	29 654 (14.3)	23 085 (16.2)	6569 (10.1)		
Family disease history					
Yes	9744 (4.7)	6005 (4.2)	3739 (5.7)	0.07	<.001^c^
No	197 825 (95.3)	136 389 (95.8)	61 436 (94.3)		

Abbreviation: SMD, standardized mean difference.

^a^ Unless otherwise indicated, data are expressed as No. (%) of patients. Percentages have been rounded and may not total 100.

^b^ Calculated using a 2-tail *t* test.

^c^ Calculated using a χ^2^ test.

^d^ Refers to the economic status that is below the local poverty line by income level.

Table 2 shows differences in violence to others during the follow-up period between the 2 groups, with the nonadherent group being more likely to perpetrate violence to others compared with adherent group, including minor nuisances (20 614 [14.5%] vs 4814 [7.4%]; *P* < .001), violating APS law (6570 [4.6%] vs 1366 [2.1%]; *P* < .001), and violating criminal law (1484 [1.0%] vs 474 [0.7%]; *P* < .001) before matching. There was a similar trend after matching. In addition, a relatively low incidence of violence to others was observed in the lowest medication adherence group (PRM < 0.2).

**Table 2.** Violence to Others During Follow-up Period

Violence	Patient group^a^						*P* value
	Medication nonadherence					Medication adherence (P5)	
	P1	P2	P3	P4	Total		
**Before matching **							
No. of patients	77 618	22 463	20 965	21 348	142 394	65 175	NA
Minor nuisances	10 561 (13.6)	3670 (16.3)	3332 (15.9)	3051 (14.3)	20 614 (14.5)	4814 (7.4)	<.001
Violating APS law	3407 (4.4)	1166 (5.2)	1025 (4.9)	972 (4.6)	6570 (4.6)	1366 (2.1)	<.001
Violating criminal law	731 (0.9)	231 (1.0)	251 (1.2)	271 (1.3)	1484 (1.0)	474 (0.7)	<.001
**After matching**							
No. of patients	27 605	9207	9224	10 319	56 355	56 355	NA
Minor nuisances	3470 (12.6)	1354 (14.7)	1387 (15.0)	1331 (12.9)	7707 (13.7)	4418 (7.8)	<.001
Violating APS law	1142 (4.1)	418 (4.5)	440 (4.8)	419 (4.1)	2468 (4.4)	1274 (2.3)	<.001
Violating criminal law	261 (0.9)	93 (1.0)	123 (1.3)	147 (1.4)	652 (1.2)	420 (0.7)	<.001

Abbreviations: APS, the Law of the People’s Republic of China on Penalties for Administration of Public Security; NA, not applicable; P1, proportion of regular medication (PRM) less than 0.20; P2, PRM 0.20 to less than 0.40; P3, PRM 0.40 to less than 0.60; P4, PRM 0.60 to less than 0.80; P5, PRM of 0.80 or more.

^a^ Unless otherwise indicated, data are expressed as No. (%) of patients.

The final DAG is shown in the Figure. The following variables were retained as confounders in our statistical models: age, sex, ethnicity, marital status, urban or rural residence, educational attainment, economic status, community case management status, duration of illness, and family history of schizophrenia (eMethods 1 in Supplement 1).

**Figure.**
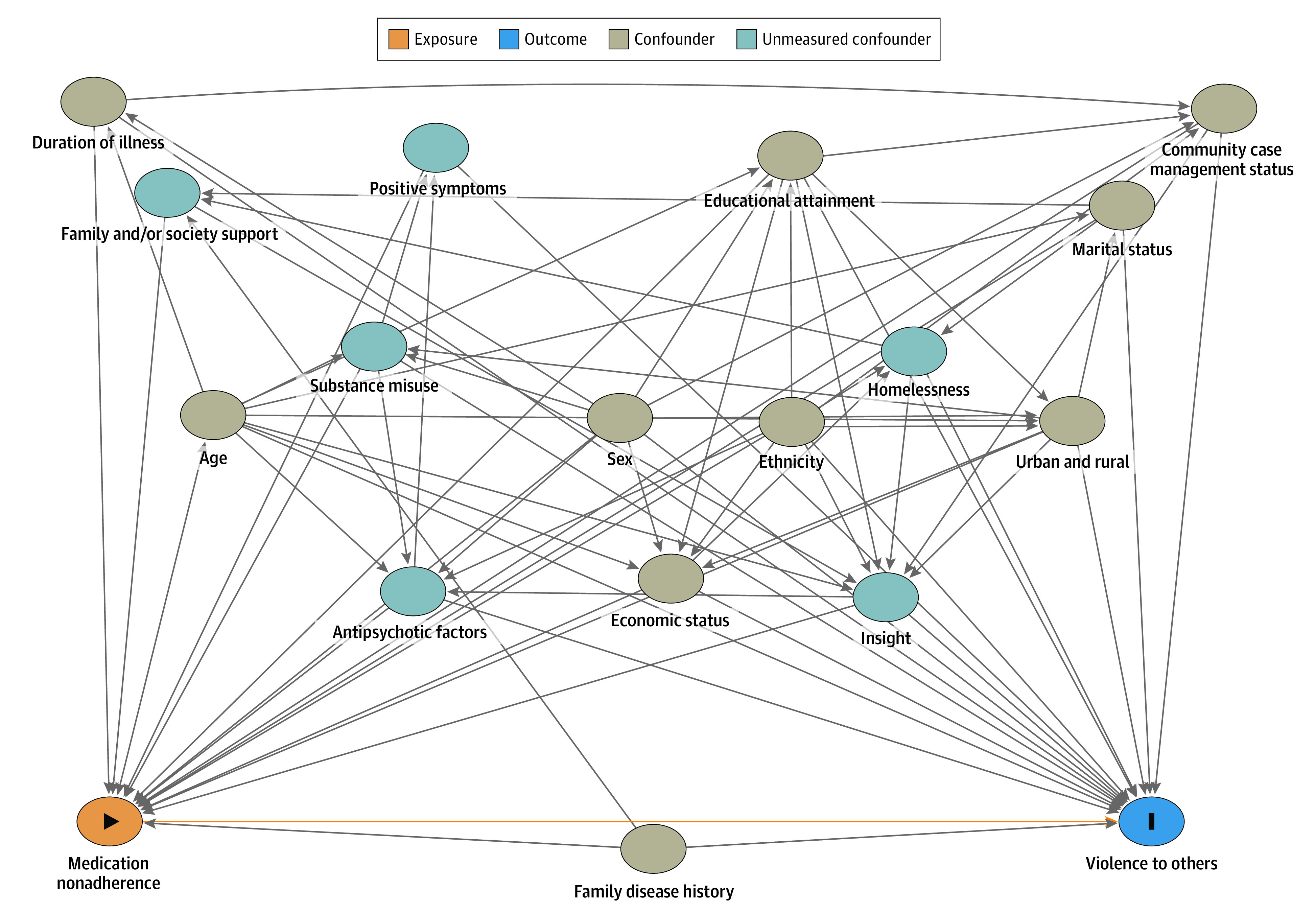
The Final Constructed Directed Acrylic Graph

Table 3 shows that medication nonadherence was associated with higher risk of minor nuisances (odds ratio [OR], 1.82 [95% CI, 1.75-1.90]; *P* < .001), violating APS law (OR, 1.91 [95% CI, 1.78-2.05]; *P* < .001), and violating criminal law (OR, 1.50 [95% CI, 1.33-1.71]; *P* < .001). In addition, regional differences in the incidence of violence to others were significant (minor nuisances: χ^2^ = 2074.15; violating APS law: χ^2^ = 2022.80; violating criminal law: χ^2^ = 1006.12; and violence to others: χ^2^ = 2371.68 [*P* < .001 for all]) (eTable 2 in Supplement 1). The range of incidence of minor nuisances was 8.2% to 25.2%; violating APS law, 1.6% to 7.6%; and violating criminal law, 0.3% to  4.5%.

**Table 3.** Association Between Medication Nonadherence and Violence to Others During Follow-up Period (for Hypothesis 1)^a^

Violence type	OR (95% CI)^b^	*z* Score	*P* value
**Minor nuisances**			
Intercept	0.09 (0.08-0.11)	−32.17	<.001
Medication nonadherence	1.82 (1.75-1.90)	29.83	<.001
**Violating APS law**			
Intercept	0.02 (0.02-0.03)	−45.08	<.001
Medication nonadherence	1.91 (1.78-2.05)	18.35	<.001
**Violating criminal law**			
Intercept	0.01 (0.01-0.01)	−31.91	<.001
Medication nonadherence	1.50 (1.33-1.71)	6.42	<.001

Abbreviations: APS, the Law of the People’s Republic of China on Penalties for Administration of Public Security; OR, odds ratio.

^a^ All models included random intercepts per region and per patient (nested within region) to account for differences between regions.

^b^ Estimates show the association of medication nonadherence with the probability of study outcomes, as calculated from generalized linear mixed-effects models and applied to a propensity score–matched data set.

Table 4 shows that all levels of medication nonadherence had higher risk of all types of violence to others compared with adherence (P5). However, the risk of violence to others did not increase with higher levels of medication nonadherence.

**Table 4.** Association Between Medication Nonadherence and Violence to Others During Follow-up Period (for Hypothesis 2)^a^

Violence type by adherence	No. of patients	OR (95% CI)^b^	*z* Score	*P* value
**Minor nuisances**				
P1 vs P5				
Intercept	86 270	0.10 (0.09-0.12)	−28.79	<.001
Medication nonadherence		1.57 (1.50-1.64)	19.77	<.001
P2 vs P5				
Intercept	44 584	0.10 (0.09-0.12)	−28.20	<.001
Medication nonadherence		2.02 (1.91-2.15)	23.38	<.001
P3 vs P5				
Intercept	41 888	0.10 (0.09-0.12)	−33.31	<.001
Medication nonadherence		1.96 (1.84-2.08)	21.61	<.001
P4 vs P5				
Intercept	42 696	0.10 (0.08-0.11)	−36.19	<.001
Medication nonadherence		1.86 (1.75-1.98)	19.55	<.001
**Violating APS law**				
P1 vs P5				
Intercept	86 270	0.03 (0.02-0.03)	−38.95	<.001
Medication nonadherence		1.66 (1.53-1.79)	12.60	<.001
P2 vs P5				
Intercept	44 584	0.03 (0.02-0.03)	−37.80	<.001
Medication nonadherence		2.03 (1.83-2.25)	13.54	<.001
P3 vs P5				
Intercept	41 888	0.03 (0.02-0.03)	−41.66	<.001
Medication nonadherence		1.94 (1.75-2.16)	12.13	<.001
P4 vs P5				
Intercept	42 696	0.02 (0.02-0.03)	−45.38	<.001
Medication nonadherence		1.99 (1.79-2.23)	12.29	<.001
**Violating criminal law**				
P1 vs P5				
Intercept	86 270	0.01 (0.01-0.01)	−28.61	<.001
Medication nonadherence		1.32 (1.14-1.53)	3.73	<.001
P2 vs P5				
Intercept	44 584	0.01 (0.01-0.01)	−28.34	<.001
Medication nonadherence		1.36 (1.11-1.66)	3.03	<.001
P3 vs P5				
Intercept	41 888	0.01 (0.004-0.01)	−27.14	<.001
Medication nonadherence		1.84 (1.49-2.27)	5.74	<.001
P4 vs P5				
Intercept	42 696	0.01 (0.01-0.01)	−34.06	<.001
Medication nonadherence		1.75 (1.44-2.15)	5.56	<.001

Abbreviations: APS, the Law of the People’s Republic of China on Penalties for Administration of Public Security; OR, odds ratio; P1, proportion of regular medication (PRM) less than 0.20; P2, PRM 0.20 to less than 0.40; P3, PRM 0.40 to less than 0.60; P4, PRM 0.60 to less than 0.80; P5, PRM of 0.80 or more.

^a^ All models included random intercepts per region and per patient (nested within region) to account for differences between regions.

^b^ Estimates show the association of medication nonadherence with the probability of study outcomes, as calculated from generalized linear mixed-effects models and applied to a propensity score–matched data set.

Subgroup analyses showed that medication nonadherence was associated with higher risk of all types of violence to others. There were significant differences in violating APS law between urban (OR, 2.61 [95% CI, 2.20-3.12]; *P* < .001) and rural (OR, 1.85 [95% CI, 1.71-1.99]; *P* < .001) areas (eTable 5 in Supplement 1). Sensitivity analyses showed basically stable results for all types of violence to others outcome in the main and subgroup analyses. Compared with minor nuisances and violating APS law, the influence of unmeasured confounders was slightly greater for violating criminal law (eTables 6-8 in Supplement 1).

## Discussion

The results of this 12-year cohort study show that patients in the community with schizophrenia and nonadherence to medicine had higher risk of minor nuisances, violating APS law, and violating criminal law. The large sample size, prospective cohort study design, and rigorous analysis techniques used to identify and control confounders all enhance the generalizability of our findings.

First, regarding the violence to others as the outcome of this study, its definition and measurement has not been a simple issue and varies in different studies.^23^ In this study, minor nuisances, violating APS law, and violating criminal law were included, and the measurements were obtained from official judicial records, which showed that a total of 27 698 (13.3%) of 207 569 patients with schizophrenia committed violence to others, of which 25 428 (12.3%) had committed minor nuisances, 7936 (3.8%) violated APS law, and 1958 (0.9%) violated criminal law. Patients who were men, living in poverty, members of an ethnic minority group, unmarried, and living in rural areas and who had less educational attainment were more likely to commit violence to others (eTable 3 in Supplement 1). These findings are consistent with the literature.^7,15,24^ In addition, those with longer duration of illness and follow-up, with a family history of psychosis, and with community case management were also more likely to commit violence to others, but these factors have not been reported and could be investigated in the future.

Second, our focus was on medication adherence. As one of the influencing factors of violence to others, there is still no criterion standard for definition and measurement of medication adherence.^25^ Existing studies often consider it as a dichotomous variable but do not provide a universally accepted cutoff point. Some clinical practice guidelines recommend adherence levels of at least 80%,^14^ which have been applied in some studies.^26,27^ In addition, a few studies consider medication adherence a continuous variable (0%-100%).^28,29^ The measurement of medication adherence includes both subjective and objective measures, with subjective measures predominating.^30^ In this study, the subjective measure was used to calculate PRM (0-1.00), which was defined as a dichotomous variable with a threshold value of 0.80. The results of this study showed that the total number of patients with nonadherence in the sample was as high as 142 394 (68.6%). Previous studies^31,32^ have shown that medication nonadherence is not consistent across studies for the same characteristics, such as age, sex, ethnicity, marital status, economic status, educational level, and duration of illness. This may be because the definition of medication nonadherence varies from study to study and that there are differences in the populations and regions in different studies.

Most important, regarding the association between medication nonadherence and violence to others, we identified only 1 study that reported medication nonadherence as the exposure.^28^ This was a 10-year prospective cohort study from Canada that recruited 11 462 individuals with schizophrenia who had committed a criminal offense; used the medication possession ratio (ranging from 0%-100%) to objectively measure medication adherence at 120-day intervals, with at least 80% defined as adherence; and took the number of violent behaviors in every 120-day interval as the primary outcome. The investigators found that lower levels of medication adherence were associated with an increased risk of crime (adjusted risk ratio, 1.58 [95% CI, 1.46-1.71]).^28^ Similarly, the present study found a higher incidence of violence to others in community-based patients with schizophrenia who were medication nonadherent, and this finding held true even after the confounders were controlled. This is generally consistent with the results of previous studies on the association between various influencing factors, including medication adherence and violence.^11,15,28^ In addition, our study found that all lower levels (P1 to P4) of adherence were associated with higher risk of violence to others compared with the reference group (P5), while there was no trend of increasing incidence of violence to others with decreasing levels of medication adherence. This result is also consistent with the findings of the aforementioned Canadian study.^28^ It is also worth noting that, contrary to our secondary hypothesis, the lowest adherence level (PRM < 0.20) was not associated with the highest violence rate. Although symptom improvement and/or remission (leading to treatment discontinuation) could explain the association between low PRM and comparatively low rates of violence to others, some studies^32,33^ suggest that antipsychotic persistence may be enhanced by effective symptom control. We observed relatively less violence to others among patients in the P1 level, which may have been the result of severe symptoms (and associated impaired function) and/or hospitalization. Alternatively, titration from medication due to symptom improvement and recovery may account for the observed association between low adherence and violence to others in these patients.^28^ Further research is needed to establish the association among levels of medication adherence, patient functioning, and violence to others.

### Limitations

This study has some limitations. First, because the outcomes and exposures were defined over the follow-up period, we can only estimate an association between medication nonadherence and violence to others, given the cross-sectional analysis design of the study. Second, due to the limitation of data accessibility, this study had the problem of unmeasured confounders, and PSM could only control for measured confounders. Although we evaluated the stability of findings using sensitivity analysis, a more accurate estimate of the effect size should be made if more comprehensive confounders can be collected in future. Third, PSM resulted in a partial loss of sample size, which limited the representativeness of sample and the inference of the results. Although this bias can be somewhat mitigated by the large sample of this study, it is still a limitation. Fourth, subjective reports were used as a measure of medication adherence in this study. Further evaluation is needed regarding the validity of this definition and objective measures should be considered to improve the accuracy. Last, there were some missing data in the variables included in this study, which we directly removed because the sample size was large enough. In the future, we may consider filling in the missing data and use the filled data to conduct sensitivity analysis to test the robustness of the results.

## Conclusions

The findings of this cohort study suggest that medication nonadherence was associated with violence to others among community-based patients with schizophrenia. These findings support the need for improving the accessibility of mental health service resources, especially in rural areas. Potential implications and future directions are found in eResults in Supplement 1.

## Notes

Supplement 1. **eTable 1.** Definition and Encoding of Variables in This Study **eFigure 1.** The Study Selection Flowchart **eTable 2.** Regional Differences of Violence to Others in Our Sample **eTable 3.** Classification of Violence to Others in Sample **eMethods 1.** The Process of Constructing Directed Acyclic Graphs (DAGs) **eMethods 2.** The Propensity Score Matching (PSM) Model Selection **eFigure 2.** Balance Check Before and After Matching for Medication Nonadherence vs Adherence in the Sample **eFigure 3.** Density Plots of the Propensity Scores in Both Groups Before and After Matching **eTable 4.** The Variance of Random Effects After Matching **eTable 5.** Subgroup Analysis of Estimated Association Between Medication Nonadherence and Violence to Others During the Follow-up Period **eMethods 3.** Rosenbaum and Rubin’s Method of Sensitivity Analysis **eTable 6.** Sensitivity Analysis of PSM Estimates With Respect to Unobserved Confounders (Main Analysis) **eTable 7.** Sensitivity Analysis of PSM Estimates With Respect to Unobserved Confounders (Subgroup Analysis by Sex) **eTable 8.** Sensitivity Analysis of PSM Estimates With Respect to Unobserved Confounders (Subgroup Analysis by Urban and Rural) **eResults.** Potential Implications of This Research and Directions for Future Study
eReferences

Supplement 2.
Data Sharing Statement
