# Supplementary material for: Medication Nonadherence and Risk of Violence to Others Among Patients With Schizophrenia in Western China
Source: JAMA Netw Open. 2023 Apr 5;6(4):e235891. doi: 10.1001/jamanetworkopen.2023.5891 (PMC10077101; doi:10.1001/jamanetworkopen.2023.5891)
Supplement: Supplement 1. — eTable 1. Definition and Encoding of Variables in This Study eFigure 1. The Study Selection Flowchart eTable 2. Regional Differences of Violence to Others in Our Sample eTable 3. Classification of Violence to Others in Sample eMethods 1. The Process of Constructing Directed Acyclic Graphs (DAGs) eMethods 2. The Propensity Score Matching (PSM) Model Selection eFigure 2. Balance Check Before and After Matching for Medication Nonadherence vs Adherence in the Sample eFigure 3. Density Plots of the Propensity Scores in Both Groups Before and After Matching eTable 4. The Variance of Random Effects After Matching eTable 5. Subgroup Analysis of Estimated Association Between Medication Nonadherence and Violence to Others During the Follow-up Period eMethods 3. Rosenbaum and Rubin’s Method of Sensitivity Analysis eTable 6. Sensitivity Analysis of PSM Estimates With Respect to Unobserved Confounders (Main Analysis) eTable 7. Sensitivity Analysis of PSM Estimates With Respect to Unobserved Confounders (Subgroup Analysis by Sex) eTable 8. Sensitivity Analysis of PSM Estimates With Respect to Unobserved Confounders (Subgroup Analysis by Urban and Rural) eResults. Potential Implications of This Research and Directions for Future Study eReferences [file jamanetwopen-e235891-s001.pdf]

Supplementary Online Content

Li Y, Wen H, Xiong C, et al. Medication nonadherence and risk of violence to others among patients with schizophrenia in western China. *JAMA Netw Open*. 2023;6(4):e235891. doi:10.1001/jamanetworkopen.2023.5891

eTable 1. Definition and Encoding of Variables in This Study
eFigure 1. The Study Selection Flowchart
eTable 2. Regional Differences of Violence to Others in Our Sample
eTable 3. Classification of Violence to Others in Sample
eMethods 1. The Process of Constructing Directed Acyclic Graphs (DAGs)
eMethods 2. The Propensity Score Matching (PSM) Model Selection
eFigure 2. Balance Check Before and After Matching for Medication Nonadherence vs Adherence in the Sample
eFigure 3. Density Plots of the Propensity Scores in Both Groups Before and After Matching
eTable 4. The Variance of Random Effects After Matching
eTable 5. Subgroup Analysis of Estimated Association Between Medication Nonadherence and Violence to Others During the Follow-up Period
eMethods 3. Rosenbaum and Rubin's Method of Sensitivity Analysis
eTable 6. Sensitivity Analysis of PSM Estimates With Respect to Unobserved Confounders (Main Analysis)
eTable 7. Sensitivity Analysis of PSM Estimates With Respect to Unobserved Confounders (Subgroup Analysis by Sex)
eTable 8. Sensitivity Analysis of PSM Estimates With Respect to Unobserved Confounders (Subgroup Analysis by Urban and Rural)
eResults. Potential Implications of This Research and Directions for Future Study
eReferences

This supplementary material has been provided by the authors to give readers additional information about their work.

© 2023 Li Y et al. *JAMA Network Open*.

eTable 1. Definition and Encoding of Variables in This Study

Variable type	Name	Definition
**Outcomes**	Minor nuisances	It refers to cases in which the public security authorities respond to the police but only for general education and other treatment, such as patients hitting, cursing others or disrupting the order, but not causing damage to life and property. It does not reach the point of violating the Law of the People's Republic of China on Penalties for Administration of Public Security.<br>No occurrence=0; Occurrence=1
	Violating APS law	It refers to acts that endanger society and harm others, such as: assault and battery causing injury, provocation, insulting women; obstructing traffic safety; snatching and damaging public and private property, etc. The acts violate the Law of the People's Republic of China on Penalties for Administration of Public Security.<br>No occurrence=0; Occurrence=1
	Violating criminal law	It refers to acts that endanger society and harm others, such as: assault and battery causing injury, provocation, insulting women; obstructing traffic safety; snatching and damaging public and private property, etc. The acts are more serious and violate the Criminal Law of the People's Republic of China.<br>No occurrence=0; Occurrence=1
	Violence-TO	Violence to others among patients with schizophrenia includes minor nuisances, violating APS law, and violating criminal law.
**Exposure**	Medication nonadherence	PRM: The proportion of regular medication during the follow-up period. Please see the details in “Exposure” in the main body of this paper.<br>Binary for hypothesis 1:<br>Adherence=0 (PRM≥0.80); Nonadherence=1 (PRM<0.80)<br>Five classification for hypothesis 2:<br>{P5=0 (PRM≥0.80) ***VS*** P4=1 (0.60≤PRM<0.80)}; {P5=0 (PRM≥0.80) ***VS*** P3=1 (0.40≤PRM<0.60)}; {P5=0 (PRM≥0.80) ***VS*** P2=1 (0.20≤PRM<0.40)}; {P5=0 (PRM≥0.80) ***VS*** P1=1 (0≤PRM<0.20)}
**Confounders**	Age,years	Age as of the last follow-up
	Sex	Women=0; Men=1
	Ethnicity	Ethnic minority=0; Han=1
	Family disease history	Family history of severe mental illness in two or three generations:<br>No = 0; Yes = 1
	Marital status	Unmarried=0; Married=1
	Urban and Rural	Rural=1; Urban=0

© 2023 Li Y et al. *JAMA Network Open*.

Educational attainment	The highest level of education obtained by the patient at the time of filing is divided into 3 levels:<br>Primary school or lower=1; Middle and high school=2; College, university, or higher=3
Economic status	Poverty refers to the economic status that is below the local poverty line by income level:<br>Non-poverty=0; Poverty=1
Follow-up time, years	Time of illness as of the last follow-up visit:<br><2 years=1; 2-3 years=2; ≥4years=3
Duration of illness, years	Total follow-up time from the first to the last visit:<br><10 years=1; 10-19=2; 20-29=3; ≥30=4
Community case management status	It indicates whether the patient participates in community case management. Community case management refers to the development and implementation of targeted phased rehabilitation programs and measures for patients who have been clearly diagnosed, based on their conditions and psychosocial functional characteristics and needs, through the assessment of their psychiatric symptoms, functional impairment or the main problems they face, so that they can receive continuous and effective treatment, recover their living and working abilities, and help them return to social life. It includes the following areas: mental health, physical health, daily life, social relationships, work and study, risk assessment, financial situation, housing situation, family guardianship and rights protection.<br>As long as case management was performed once during a particular follow-up period, the patient was considered to have been case managed for the entire follow-up period.<br>None=0; Yes=1

© 2023 Li Y et al. *JAMA Network Open*.

eFigure 1. The Study Selection Flowchart

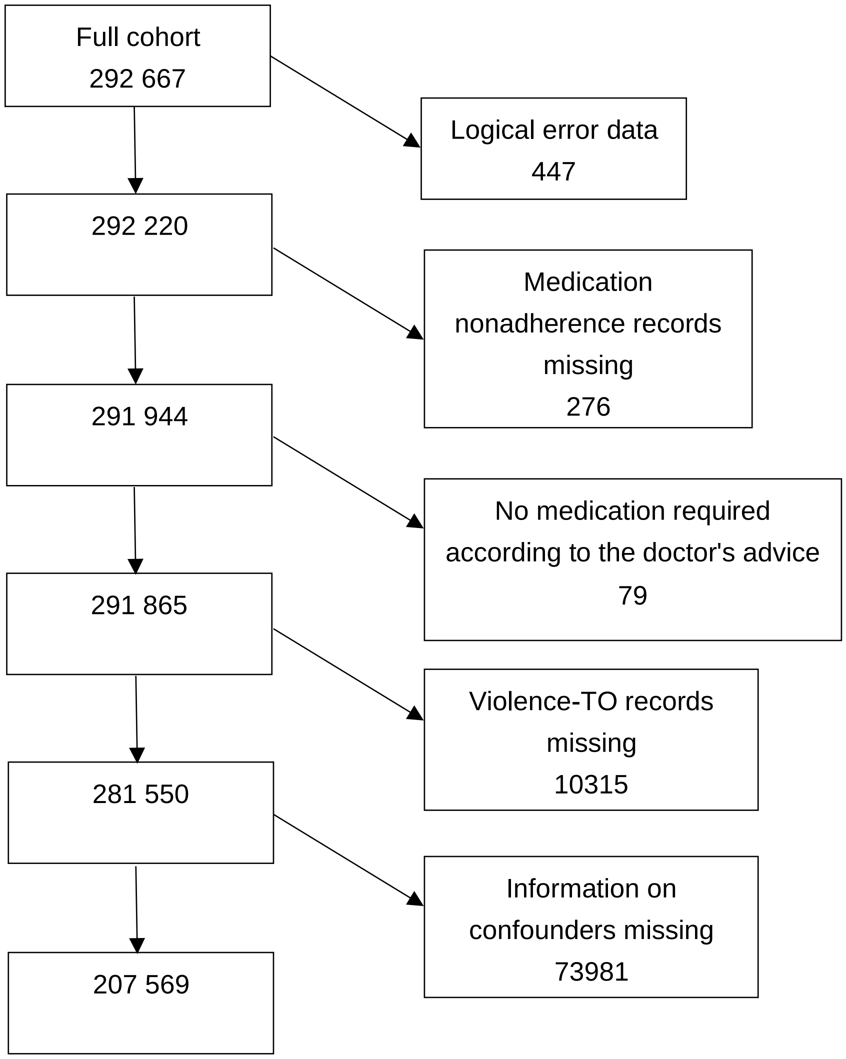

© 2023 Li Y et al. *JAMA Network Open*.

eTable 2. Regional Differences of Violence to Others in Our Sample

Regions	n	Minor nuisances, No.(%)	Violating APS law, No.(%)	Violating criminal law, No.(%)	Violence-TO, No.(%)
1	33952	2801 (8.2)	683 (2.0)	133 (0.4)	2996 (8.8)
2	10892	1365 (12.5)	421 (3.9)	277 (2.5)	1646 (15.1)
3	2600	379 (14.6)	103 (4.0)	35 (1.3)	421 (16.2)
4	12992	1128 (8.7)	343 (2.6)	76 (0.6)	1223 (9.4)
5	8008	1161 (14.5)	284 (3.5)	36 (0.4)	1248 (15.6)
6	11646	1815 (15.6)	647 (5.6)	93 (0.8)	1948 (16.7)
7	6637	1139 (17.2)	473 (7.1)	72 (1.1)	1211 (18.2)
8	7963	868 (10.9)	245 (3.1)	38 (0.5)	920 (11.6)
9	12418	1615 (13.0)	628 (5.1)	71 (0.6)	1781 (14.3)
10	6745	862 (12.8)	295 (4.4)	56 (0.8)	934 (13.8)
11	17585	2863 (16.3)	976 (5.6)	285 (1.6)	3060 (17.4)
12	8042	660 (8.2)	125 (1.6)	34 (0.4)	723 (9.0)
13	12137	1267 (10.4)	379 (3.1)	134 (1.1)	1452 (12.0)
14	10037	1242 (12.4)	381 (3.8)	43 (0.4)	1331 (13.3)
15	16726	2160 (12.9)	597 (3.6)	333 (2.0)	2449 (14.6)
16	3223	382 (11.9)	124 (3.8)	39 (1.2)	432 (13.4)
17	10516	1848 (17.6)	740 (7.0)	79 (0.8)	1930 (18.4)
18	11079	904 (8.2)	256 (2.3)	30 (0.3)	979 (8.8)
19	618	156 (25.2)	47 (7.6)	28 (4.5)	165 (26.7)
20	366	65 (17.8)	13 (3.6)	9 (2.5)	70 (19.1)
21	3387	748 (22.1)	176 (5.2)	57 (1.7)	779 (23.0)

χ^2 test uesd to compare regional differences in the incidence of Minor nuisances(χ^2=2074.15；P<0.001),Violating APS law(χ^2=2022.80；P<0.001),Violating criminal law(χ^2=1006.12；P<0.001),violence to others(χ^2=2371.68；P<0.001).

© 2023 Li Y et al. *JAMA Network Open*.

eTable 3. Classification of Violence to Others in Sample

	Total cohort	No violence-TO, No.(%)	Violence-TO, No.(%)	P value
	207569	179871(86.7)	27698(13.3)	
**Exposure**				
Medication nonadherence				<0.001
Adherence	65175	59789(33.2)	5386(19.4)	
Nonadherence	142394	120082(66.8)	22312(80.6)	
PRM,mean(SD)	0.47 (0.40)	0.48(0.40)	0.39(0.35)	<0.001
**Confounders**				
Age,mean(SD),years	51.3(14.5)	51.3(14.5)	51.4(14.0)	0.18
Sex				<0.001
Men	100298	85797(47.7)	14501(52.4)	
Women	107271	94074(52.3)	13197(47.6)	
Ethnicity				<0.001
Han	205045	177847(98.9)	27198(98.2)	
Ethnic minority	2524	2024(1.1)	500(1.8)	
Marital status				<0.001
Married	134154	117279(65.2)	16875(60.9)	
Unmarried	73415	62592(34.8)	10823(39.1)	
Urban and Rural				<0.001
Rural	170534	145908(81.1)	24626(88.9)	
Urban	37035	33963(18.9)	3072(11.1)	
Educational attainment				<0.001
Primary school or lower	140357	120388(66.9)	19969(72.1)	
Middle and high school	63688	56181(31.2)	7507(27.1)	
College,university, or higher	3524	3302(1.8)	222(0.8)	
Economic status				<0.001
Poverty [a]	135868	115278(64.1)	20590(74.3)	
Non-poverty	71701	64593(35.9)	7108(25.7)	
Community case management status				<0.001
Yes	206758	179125(99.6)	27633(99.8)	
No	811	746(0.4)	65(0.2)	
Follow-up time,years				<0.001
<2	47329	44898(25.0)	2431(8.8)	
2-3	61596	56289(31.3)	5307(19.2)	
≥4	98644	78684(43.7)	19960(72.0)	
Duration of illness,years				<0.001
<10	72962	65983(36.7)	6979(25.2)	
10-19	65178	55276(30.7)	9902(35.7)	
20-29	39775	33549(18.7)	6226(22.5)	
≥30	29654	25063(13.9)	4591(16.6)	
Family disease history				0.02
Yes	9744	8364(4.6)	1380(5.0)	
No	197825	171507(95.4)	26318(95.0)	

© 2023 Li Y et al. *JAMA Network Open*.

Abbreviations: PRM, Percentage of Regular Medication. Nonadherence defined as PMR <0.8.[a] Refers to the economic status that is below the local poverty line by income level.Percentages have been rounded and may not total 100.

eMethods 1. The Process of Constructing Directed Acyclic Graphs (DAGs)

The DAG is a graphical model that describes a set of hypotheses of causal processes and is able to generate the variables we are interested in. The arrow X → Y indicates a direct causal effect of X on Y. The presence of an arrow represents the presence of causality for at least one member of the population and its direction, while the absence of an arrow represents the strong hypothesis of no causal relationship for any member of the population.[1]

Our DAG was built based on the protocol of “Evidence Synthesis for Constructing Directed Acyclic Graphs” (ESC-DAGs), which combined evidence synthesis strategies and causal inference principles. [2] First, we determined a pool of potential confounding according to systematic literature review. Second, we assumed a saturated DAG by drawing directed or undirected edges between all variables, i.e., assuming that there was causal association between each pair of exposure, outcome and confounding factors. Third, each edge in the saturated DAG was assessed using several causal criteria (including temporality, validity, and theoretical support) and determined as retained, reversed, deleted. Fourth, a simplified DAG was constructed, thereby a series of conditional independences were generated according to the constructed DAG. Lastly, we continuously did the independence test and modified the DAG if the conditional independence did not agree with our data, until all the implied conditional independences were satisfied and the final DAG was reached.

© 2023 Li Y et al. *JAMA Network Open*.

eMethods 2. The Propensity Score Matching (PSM) Model Selection

The matched models included age squared , to account for possible curvilinear relationships and were performed 1:1 nearest neighbour matching approach and a caliper set at 0.2 standard deviation of the logit of the propensity score and no-interaction was used to match medication non-adherent and adherent patients according to similar propensity score values that resulted in the good covariate balance.

eFigure 2 summarizes the balance of the covariates before and after propensity score matching (PSM). The covariate balance is the standardized mean difference as a percentage of the average standard deviation as defined by Rosenbaum and Rubin,if the absolute value of standardize mean difference is greater than 0.1, it means there is serious imbalance of the covariate between these two groups [3-4].

eFigure 3 shows the density plots of the propensity scores in both groups before and after matching and matching procedure resulted in nice propensity score overlap between two groups.

© 2023 Li Y et al. *JAMA Network Open*.

eFigure 2. Balance Check Before and After Matching for Medication Nonadherence vs Adherence in the Sample

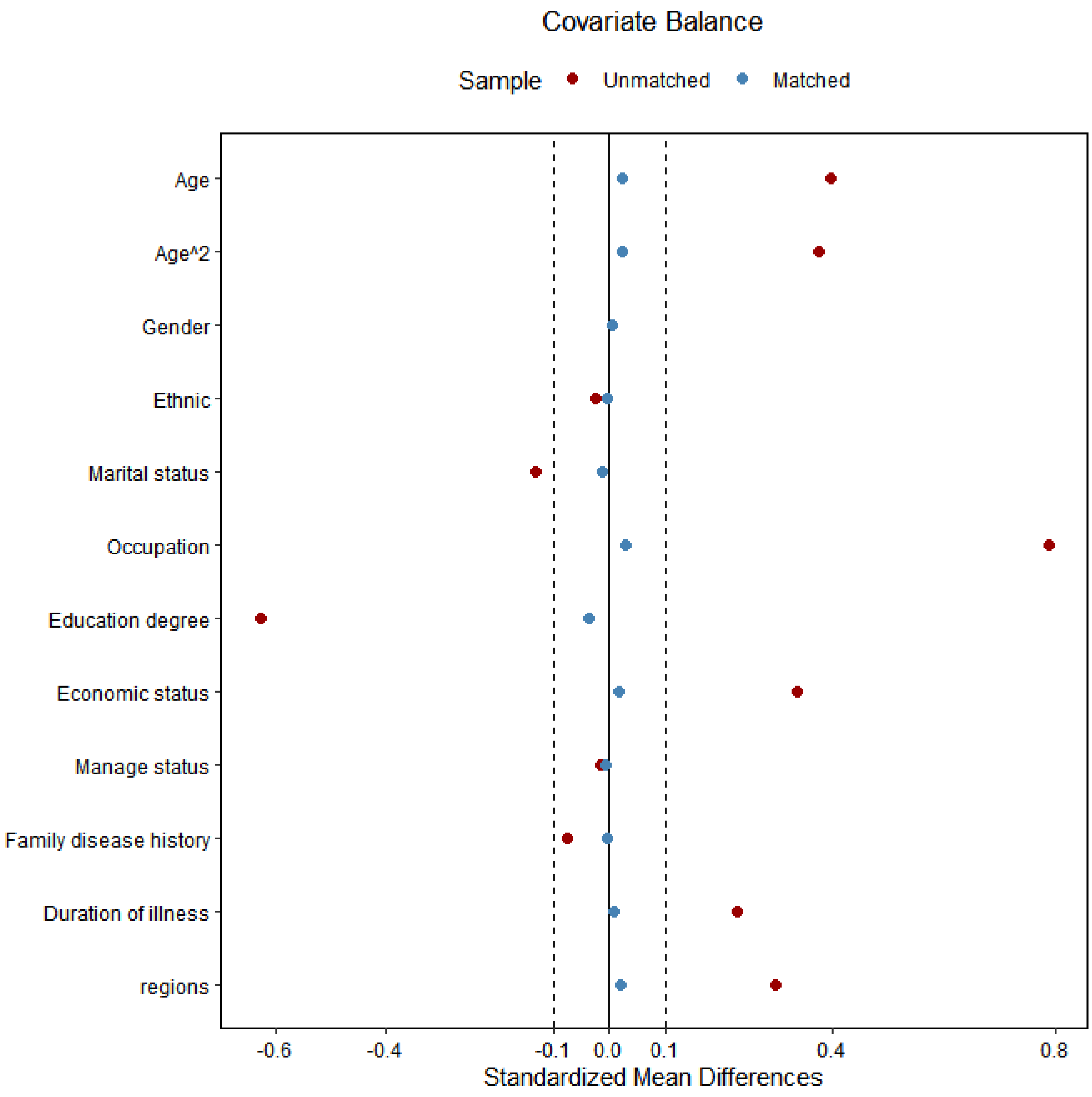

Overall, matching reduces imbalance between groups. Red circles denote values before matching and blue circles denote values after propensity score matching.

© 2023 Li Y et al. *JAMA Network Open*.

eFigure 3. Density Plots of the Propensity Scores in Both Groups Before and After Matching

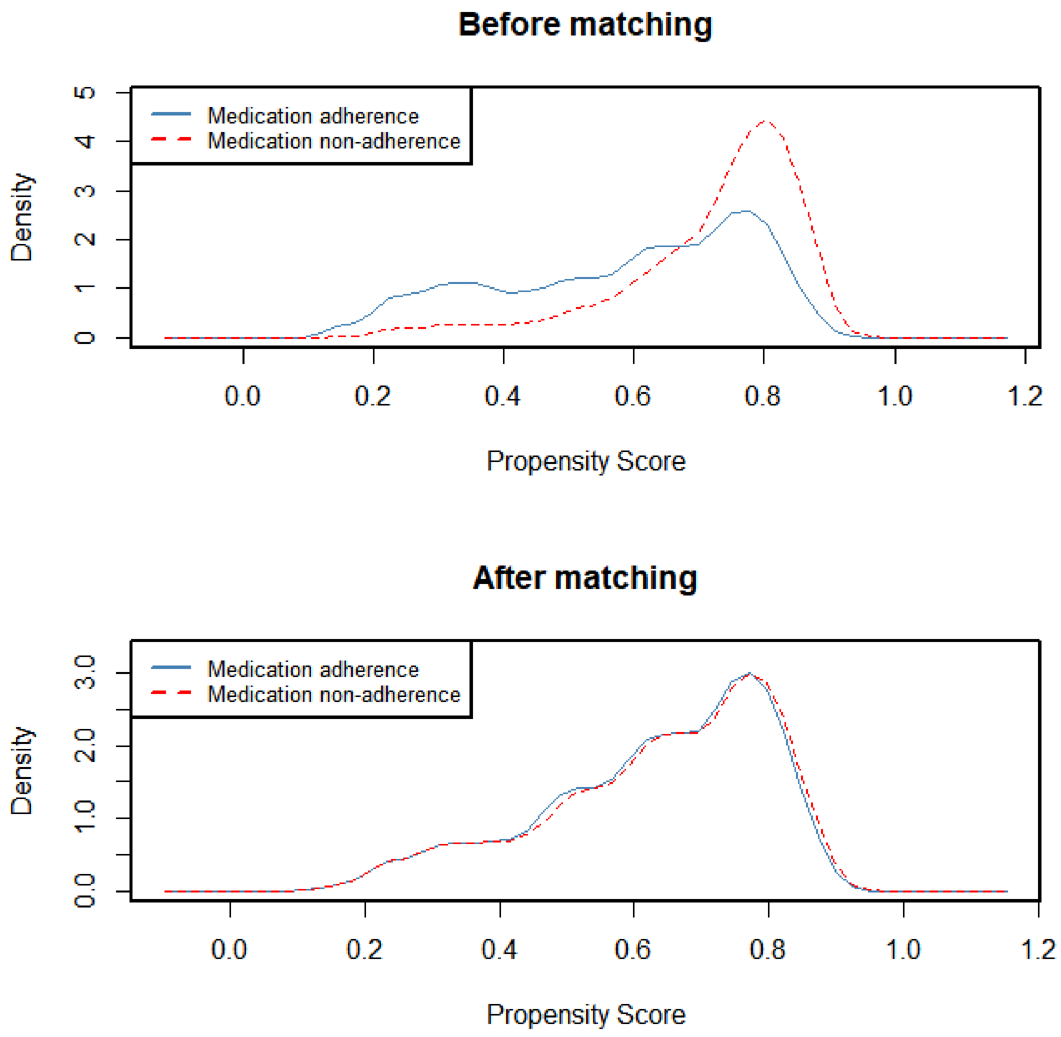

Density plots of propensity score distributions of controls (blue solid line) and comparison groups (broken red line) before and after matching.

© 2023 Li Y et al. *JAMA Network Open*.

eTable 4. The Variance of Random Effect After Matching

Random effect	Estimate (95% CI)	*Z*	P value
**Minor nuisances**			
Var (Intercept)	0.110(0.057-0.213)	2.97	0.01
**Violating APS law**			
Var (Intercept)	0.131(0.067-0.258)	2.90	0.01
**Violating criminal law**			
Var (Intercept)	0.464(0.229-0.940)	2.78	0.01

The null model showed that the variance of the random effects intercept was statistically significant, suggesting that there was still a hierarchical structure of data.

© 2023 Li Y et al. *JAMA Network Open*.

eTable 5. Subgroup Analysis of Estimated Association Between Medication Nonadherence and Violence to Others During the Follow-up Period

Violence-TO in subgroups	n	Odds ratio [a] (95%	*Z*	p value
**Sex**				
Minor nuisances				
Men	6194	1.766(1.672-1.866)	20.32	<0.001
Women	5734	1.859(1.756-1.968)	21.37	<0.001
Violating APS law				
Men	2053	1.613(1.364-1.912)	12.47	<0.001
Women	1603	2.027(1.824-2.255)	13.10	<0.001
Violating criminal law				
Men	578	1.613(1.363-1.912)	5.57	<0.001
Women	448	1.404(1.162-1.699)	3.53	<0.001
**Urban and Rural**				
Minor nuisances				
Rural	9670	1.776(1.700-1.856)	25.66	<0.001
Urban	2060	2.202(1.820-2.001)	14.30	<0.001
Violating APS law				
Rural	3028	1.847(1.712-1.994)	15.79	<0.001
Urban	634	2.612(2.195-3.122)	10.74	<0.001
Violating criminal law				
Rural	781	1.513(1.310-1.750)	5.65	<0.001
Urban	258	1.726(1.340-2.235)	4.23	<0.001

[a] Odds ratio estimates show the effect of medication nonadherence on the probability of study outcomes, as calculated from generalised linear mixed-effects models, and applied to a propensity score-matched dataset.

© 2023 Li Y et al. *JAMA Network Open*.

eMethods 3. Rosenbaum and Rubin's Method of Sensitivity Analysis

Rosenbaum's method of sensitivity analysis is based on parameter *Γ* that measures the degree of departure from random group assignment after matching. Two subjects with the same observed confounders might differ in the odds of being assigned to the "treated" group (group code = 1) by a factor of *Γ* ,[5]which is defined as the formula below, where P1/(1-P1) is the odd of being a subject in the "treated" group, whereas P0/(1-P0) is the odd of being a corresponding matching subject from the "control" group (group code = 0).

$$\frac{1}{\Gamma} \leq \frac{P1/(1-P1)}{P0/(1-P0)} \leq \Gamma$$

In a randomized experiment, randomized group assignment ensures that *Γ* = 1. In an observational study, if *Γ* = 2, then two subjects who are identical on matched confounders would have one gotten twice possibility of being in a "treated" group compared to the other, because of unobserved confounders.[5] While values of *Γ* are unknown, we can try several values of *Γ* and see if the conclusions (critical *p*-values based on function to calculate Rosenbaum bounds for binary data.) of the study change. Thus, Rosenbaum's method is meaningful to examine to what extent the significant group difference might change by unobserved confounders, in order to gauge how robust the significance testing is, based on the available matched samples. It has been suggested that when the group difference is significant after matching and the upper bound on the *p*-value is less than 0.05 for reasonably large values of *Γ* (around 1.5 to 2 in most cases), then the group difference could be considered to be robust to hidden bias.[5] The sensitivity analyses were conducted using R version 4.2.1 rbounds[6] package.

eTable 6-8 showed that for the two violent-To outcomes of minor nuisances and violating APS law, the sensitivity analysis results of main analysis and subgroup analysis showed that when the unobserved confounders makes *Γ* >1.7, which corresponds to the probability of being in the exposed group being approximately 0.63 or 0.37, rather than 0.5 (Conditional independence holds,*Γ* =1),and our conclusion is unstable（the upper bound *p* > 0.05）,which is less likely to happen. For the violating criminal law，it showed that when *Γ* >1.3, which corresponds to the probability of being in the exposed group being approximately 0.57 or 0.43, and our conclusion is unstable（the upper bound *p* > 0.05.The felony is also stable when there is no unmeasured confounding with large effects. However, considering limited sample size in the violating criminal law, it is possible that with the inclusion of more samples, such an effect would be more robust. In addition, violating criminal law were more susceptible to unmeasured confounding than minor nuisances and violating APS law.

eTable 6. Sensitivity Analysis of PSM Estimates With Respect to Unobserved Confounders (Main Analysis)

Γ [a]	**Minor nuisances**	**Violating APS law**	**Violating criminal law**
**1.0**	0.000	0.000	0.000
**1.1**	0.000	0.000	0.000
**1.2**	0.000	0.000	0.000
**1.3**	0.000	0.000	0.014
**1.4**	0.000	0.000	0.157
**1.5**	0.000	0.000	0.534
**1.6**	0.000	0.000	0.865
**1.7**	0.000	0.000	0.980
**1.8**	0.260	0.018	0.998
**1.9**	0.979	0.286	0.999
**2.0**	1.000	0.814	1.000

Abbreviations: PSM, propensity score matching.

[a] *Γ* is Odds of differential assignment to treatment due to unobserved confounders. Rosenbaum Bounds(*P*-value associated with upper bound).

© 2023 Li Y et al. *JAMA Network Open*.

eTable 7. Sensitivity Analysis of PSM Estimates With Respect to Unobserved Confounders (Subgroup Analysis by Sex)

Γ [a]	Men (n = 53 822)			Women (n = 58 764)		
	**Minor nuisances**	**Violating APS law**	**Violating criminal**	**Minor nuisances**	**Violating APS law**	**Violating criminal law**
1.0	0.000	0.000	0.000	0.000	0.000	0.000
1.1	0.000	0.000	0.000	0.000	0.000	0.004
1.2	0.000	0.000	0.000	0.000	0.000	0.040
1.3	0.000	0.000	0.008	0.000	0.000	0.188
1.4	0.000	0.000	0.062	0.000	0.000	0.681
1.5	0.000	0.000	0.234	0.000	0.000	0.881
1.6	0.000	0.003	0.511	0.000	0.000	0.967
1.7	0.065	0.072	0.767	0.001	0.000	0.993
1.8	0.700	0.408	0.918	0.154	0.003	0.998
1.9	0.992	0.822	0.978	0.801	0.040	0.999
2.0	0.999	0.978	0.995	0.995	0.213	1.000

Abbreviations: PSM, propensity score matching.

[a] Γ is Odds of differential assignment to treatment due to unobserved confounders. Rosenbaum Bounds (*P*-value associated with upper bound)

© 2023 Li Y et al. *JAMA Network Open*.

eTable 8. Sensitivity Analysis of PSM Estimates With Respect to Unobserved Confounders (Subgroup Analysis by Urban and Rural)

Γ [a]	Rural(n = 85 194)			Urban(n = 26 176)		
	Minor nuisances	Violating APS law	Violating criminal law	Minor nuisances	Violating APS law	Violating criminal law
1.0	0.000	0.000	0.000	0.000	0.000	0.000
1.1	0.000	0.000	0.000	0.000	0.000	0.000
1.2	0.000	0.000	0.002	0.000	0.000	0.002
1.3	0.000	0.000	0.047	0.000	0.000	0.012
1.4	0.000	0.000	0.255	0.000	0.000	0.049
1.5	0.000	0.000	0.613	0.000	0.000	0.134
1.6	0.000	0.000	0.879	0.000	0.000	0.274
1.7	0.013	0.008	0.977	0.005	0.000	0.448
1.8	0.621	0.176	0.997	0.085	0.000	0.622
1.9	0.996	0.679	0.999	0.405	0.000	0.766
2.0	1.000	0.962	1.000	0.796	0.001	0.869

Abbreviations: PSM, propensity score matching.

[a] Γ is Odds of differential assignment to treatment due to unobserved confounders. Rosenbaum Bounds (*P*-value associated with upper bound)

© 2023 Li Y et al. *JAMA Network Open*.

eResults. Potential Implications of This Research and Directions for Future Study

In future, relevant government departments should carry out structural optimization to make treatment services more accommodating and accessible in order to improve the accessibility of mental health service resources, especially in rural areas. Medical institutions at all levels should improve mental health management systems to enhance the identification and intervention of patients at high risk of violence, thereby achieving early prevention and control of violent behavior. Meanwhile, more objective assessment of medication nonadherence should be incorporated into the management system for schizophrenics in community. Evidence of risk of violence-TO in patients with different level of medication adherence, especially the lowest one, needs to be explored in future studies. Community case management of the patients should be enhanced in the future to minimize medication nonadherence.

© 2023 Li Y et al. *JAMA Network Open*.

eReferences

1. Elwert F. Graphical causal models. In: Morgan SL, ed. Handbook of Causal Analysis for Social Research. New York: Springer; **2013**: 245-73.

2. Lewsey JD, Smith DJ, Green MJ, et al. Evidence synthesis for constructing directed acyclic graphs (ESC-DAGs): a novel and systematic method for building directed acyclic graphs[J]. Int J Epidemiol 2020, **49(1)**:322-329.

3. Rosenbaum PR, Rubin DB. Constructing a control-group using multivariate matched sampling methods that incorporate the propensity score. Am Stat. 1985;**39(1)**:33-38.

4. Rosenbaum PR, Rubin DB. The central role of the propensity score in observational studies for causal effects. Biometrika. 1983;**70(1)**:41-55.

5. Rosenbaum PR. Observational Studies. Vol 2nd ed. New York, NY: Springer; 2002.

6. Keele L. An overview of rbounds: An R package for Rosenbaum bounds sensitivity analysis with matched data. August 2010.

© 2023 Li Y et al. *JAMA Network Open*.
