# Supplementary material for: Medication Nonadherence and Risk of Violence to Others Among Patients With Schizophrenia in Western China
Source: JAMA Netw Open. 2023 Apr 5;6(4):e235891. doi: 10.1001/jamanetworkopen.2023.5891 (PMC10077101; doi:10.1001/jamanetworkopen.2023.5891)
Supplement: Supplement 2. — Data Sharing Statement [file jamanetwopen-e235891-s002.pdf]

Data Sharing Statement

Li. Medication Nonadherence and Risk of Violence to Others Among Patients With Schizophrenia in Western China. *JAMA Netw Open*. Published April 05, 2023. doi:10.1001/jamanetworkopen.2023.5891

Data
Data available: No

Additional Information
Explanation for why data not available: The data cannot be shared because this data involves patient details and we have a confidentiality agreement with the authorities responsible for the data and cannot share the data.
